# Giant cell tumor of soft tissue: GCT arising from periosteum of tibia

**DOI:** 10.12669/pjms.40.2(ICON).8959

**Published:** 2024-01

**Authors:** Syed Kamran Ahmed, Muhammad Nirman Shehzad

**Affiliations:** 1 Prof. Dr. Syed Kamran Ahmed, FCPS. Department of Orthopedics, The Indus Hospital and Health Network, Karachi, Pakistan; 2 Dr. Muhammad Nirman Shehzad, FCPS. Department of Orthopedics, The Indus Hospital and Health Network, Karachi, Pakistan

**Keywords:** Soft Tissue GCT (GCT-ST), Periosteum of bone

## Abstract

Primary Giant Cell Tumor of Soft Tissue (GCT-ST) is a rare disease, a neoplasm with low potential for malignancy. It belongs to the group of Fibrohistiocytic tumors with borderline malignancy. Most commonly it presents as a painless, slow-growing mass in a superficial location. It is associated with lower local recurrence rate as compared to GCT of bone but has a higher rate for metastasis and mortality. A case of rare GCT-ST with suspicion of lung metastasis is being reported here. The lesion per-operatively appeared to be growing from the periosteum of the bone (tibia in our case). After excisional biopsy it proved to be GCT-ST which has never been reported previously in literature.

## INTRODUCTION

Giant cell tumor of soft tissue (GCT-ST) is a rare primary neoplasm that usually presents as a painless mass in the extremities and trunk. It is a defined disease that has a morphology similar to the Giant Cell Tumor of Bone and is composed of mononuclear and osteoclast-like multinucleated giant cell. It typically occurs in the fifth decade of life and requires histology and immunochemistry for diagnosis. Treatment usually involves marginal resection of the lesion. It is considered to be of malignant potential; therefore, close clinical follow-up is recommended.^1^

## CASE REPORT

A 34 year old male patient with no previously reported medical issues, presented with a painful localized swelling over the anterior aspect of right leg. The swelling was noticed by the patient five years ago following a minor trauma, but became painful six months back which was the reason to seek medical care.

Examination revealed a tender, soft to firm mass over the antero-medial aspect of right leg measuring about 2 x 3 cm in size. It was adherent to the underlying structure, not freely mobile. The overlying skin could be pinched. MRI showed an abnormal signal intensity lesion involving the subcutaneous tissues of the mid leg antero-medially, measuring 1.4 x 1.0 cm. It was iso-intense on T1 weighted images, heterogeneous on T2 weighted images, heterogeneously hyper-intense on fat-suppressed images and showed heterogeneous post contrast enhancement. There was mild adjacent fat stranding.

Excisional Biopsy was done. Pre-operative staging was not performed as the lesion, clinically and radiologically, appeared to be of benign type. Marginal excision of the mass was performed along with the underlying periosteum of tibia. Microscopic examination showed multinodular sheets of compactly arrange mononuclear spindle cells along with osteoclast like multinucleated giant cells. Mononuclear cells were spindle shaped with eosinophilic cytoplasm. Peripheral rim of metaplastic bone formation with extension into the lesion. 1-2 mitosis /10 HPF noted. No evidence of nuclear pleomorphism, sarcomatoid change noticed. Immunohistochemistry showed ASMA to be patchy positive, CD68 positive in multinucleated giant cells. S-10 and Desmin was negative. The margins were free of tumor. The lesion did not appear to have bone origin and hence a diagnosis of Primary soft tissue Giant cell tumor was established. Computed Tomography of the chest was done for Lung metastasis, which reported small/ tiny soft tissue nodules only conspicuous on MIP images which are too small to be characterized. MRI repeated at 01year follow-up showed no signs of local recurrence. Chest nodule remained constant in size on repeat scan.


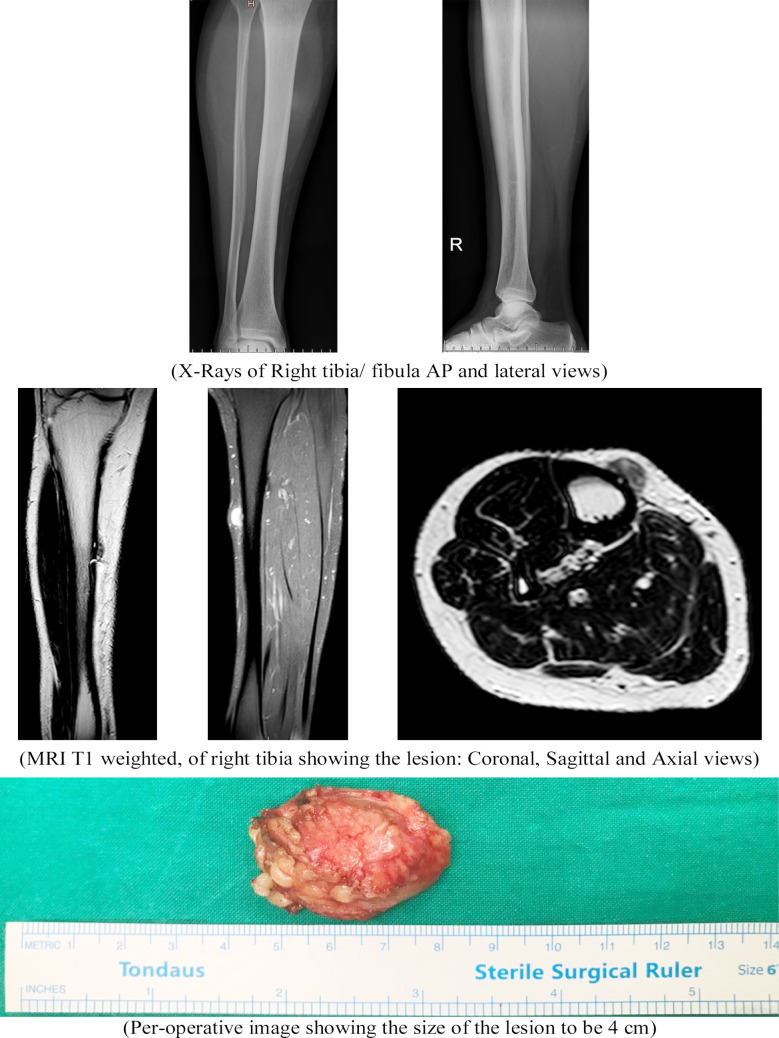


## DISCUSSION

Giant Cell Tumor of Soft Tissue (GCT-ST) is a rare neoplasm, which was first described by Salm and Sisson.^2^ They can present as well-circumscribed multinodular masses covered by normal skin or with a fleshy red-brown surface when superficially located.^3^ Soft-tissue GCTs can have a clinical presentation that mimics other soft tissue masses, such as hematoma or musculoskeletal tumors. They can appear as cystic masses on imaging and may be mistaken for organizing hematomas and hence histopathological evaluation is necessary to confirm the diagnosis.^4^,^5^ It is considered to have a low malignant potential,^6^ however it may carry a high metastasis and death rate as described in a few studies.^1^ In our case the lesion was arising from the periosteum of tibia. It was excised en bloc along with the periosteum. We did not find any case report in literature with GCT-ST arising from the periosteum of the bone. Since the literature suggests a higher distance metastasis rate and our patient had a suspicious lung metastasis to begin with, we are keeping close follow up of the patient to ensure timely treatment in case any new findings develop.

### Authors’ Contribution:

**SKA and MNS** conceived, designed, critically reviewed, edited manuscript, and are responsible for integrity of the research.
